# Bone health and physical activity in adolescents with juvenile idiopathic arthritis: a cross-sectional case-control study

**DOI:** 10.1186/s12969-024-00982-4

**Published:** 2024-04-19

**Authors:** Egi Vasil, Colleen M. Nesbitt, Clodagh Toomey, Gregor Kuntze, Shane Esau, Carolyn A. Emery, Leigh Gabel

**Affiliations:** 1 Human Performance Laboratory, Faculty of Kinesiology, University of Calgary, 2500 University Dr NW, T2N 1N4 Calgary, AB Canada; 2 McCaig Institute for Bone and Joint Health, University of Calgary, Calgary, Canada; 3 Sport Injury Prevention Research Center, Faculty of Kinesiology, University of Calgary, Calgary, Canada; 4 School of Allied Health, Faculty of Education and Health Sciences, University of Limerick, Limerick, Ireland; 5 Alberta Bone and Joint Health Institute, Calgary, AB Canada; 6 Departments of Community Health Sciences and Paediatrics, Cumming School of Medicine, University of Calgary, Calgary, Canada; 7 Alberta Children’s Hospital Research Institute, University of Calgary, Calgary, Canada

**Keywords:** Juvenile idiopathic arthritis, Bone, Physical activity, Lean mass

## Abstract

**Background:**

Adolescents with juvenile idiopathic arthritis (JIA) tend to engage in less physical activity than their typically developing peers. Physical activity is essential for bone development and reduced physical activity may detrimentally effect bone health. Thus, we examined differences in total body bone mineral content (BMC) and areal bone mineral density (aBMD) between adolescents with JIA and adolescent controls without JIA. We also examined associations between moderate-to-vigorous physical activity (MVPA), lean mass, and bone outcomes.

**Methods:**

Participants included 21 adolescents with JIA (14 females, 7 males) and 21 sex- and age-matched controls aged 10–20 years. Assessments included: height; weight; triple-single-leg-hop distance (TSLH); MVPA by accelerometry; and total body BMC, aBMD, and lean mass measured using dual X-ray absorptiometry. Height-adjusted z-scores were calculated for BMC and aBMD and used for all analyses. Multiple linear mixed effects models examined group differences in BMC and aBMD, adjusting for sex, maturity, MVPA, TSLH, and lean mass. Participants clusters, based on sex and age (within 18 months), were considered random effects.

**Results:**

Adolescents with JIA had lower total body aBMD z-scores [β (95% CI); -0.58 (-1.10 to -0.07), *p* = 0.03] and BMC z-scores [-0.47 (-0.91 to -0.03), *p* = 0.04] compared with controls. Mean daily MVPA was 22.0 min/day lower in adolescents with JIA than controls; however, MVPA was not associated with aBMD [-0.01 (-0.01 to 0.01), *p* = 0.32] or BMC [0.00 (-0.01 to 0.00), *p* = 0.39]. Lean mass was positively associated with aBMD [0.05 (0.01 to 0.09) g/cm^2^, *p* = 0.03] and BMC [0.06 (0.03 to 0.10) g, *p* < 0.001].

**Conclusion:**

Adolescents with JIA had lower total body aBMD and BMC compared with sex- and age-matched controls without JIA. Group differences in bone outcomes were not associated with the lower MVPA participation of adolescents with JIA. Despite this, physical activity should still be encouraged as it promotes physical well-being.

## Introduction

Juvenile idiopathic arthritis (JIA) is an autoimmune disease acquired during childhood. JIA results from a disturbed balance between proinflammatory effector cells and anti-inflammatory regulating cells [1]. In Canada, 1 in 1000 children suffer from JIA which affects 0.07–4.01 per 1000 youth worldwide [2]. Children with JIA suffer from a range of symptoms including joint pain and swelling which can make it difficult to complete daily activities of living [1]. Youth with JIA may find it difficult to use the stairs, sit for long periods of time, and play outside due to pain [3]. The joint pain and swelling that JIA causes reduces range of motion which can result in reduced physical activity participation [4–7]. Youth with JIA have also reported hesitating to participate in physical activity as they believe it will be painful and others may judge their reduced ability [3]. Common treatments for JIA include various anti-rheumatic drugs that seek to reduce inflammation and symptoms [8–12]. Effectiveness of drugs varies between individuals and not all types of JIA respond positively to drug therapies [8–12]. Physical activity and exercise are important non-pharmacological treatments for JIA that help build bone and muscle [13, 14] and are used in conjunction with pharmacological therapies to treat individuals with JIA.

Childhood onset of arthritis has been shown to increase fracture incidence by 1.5-4.0 times that of non-arthritic healthy controls across the lifespan [15]. Since children and adolescents with JIA are less likely to engage in recommended levels of physical activity compared with their healthy peers [16, 17], they are at greater risk of compromised bone health. Weight-bearing physical activity during the critical periods of childhood and adolescence is important for optimal bone mass accrual [18, 19] and is positively associated with total body bone mineral content (BMC) in both children with JIA and typically developing (TD) youth [8]. Physical activity and exercise are promising therapies for managing JIA symptoms and improving bone health.

Accrual and consolidation of bone mineral density (BMD) is mediated by lean mass as muscle transmits forces to bone [20]. In a two-year longitudinal study, children and adolescents with JIA performed significantly less self-reported leisure time weight bearing physical activity and had less gains in lean mass and BMC compared to TD controls [13]. Weight bearing physical activity was significantly associated with increases in total body BMC in both children with JIA and TD children [13]. While supervised weight bearing exercise interventions have proved beneficial in improving quality of life and bone health in youth with JIA [4, 13], a recent study found that an at home exercise intervention had low adherence and minimal effect on bone mass, structure, and strength [21]. By better understanding the factors that are associated with poor bone health in children and adolescents with JIA, including physical activity, we may be able to develop programs to improve their bone health.

The primary aim of this study is to examine differences in BMC and areal BMD (aBMD) between adolescents with JIA and healthy adolescents. The secondary aim is to determine the relationship between free-living physical activity and bone outcomes and lean mass and bone outcomes. We hypothesize that adolescents with JIA will have significantly reduced BMC and aBMD compared with their TD peers. We further expect that adolescents engaging in more moderate to vigorous physical activity (MVPA) will have greater BMC and aBMD.

## Methods

### Study design

This is a secondary analysis of previously collected cross-sectional data [22]. Ethics approval was granted by the University of Calgary Conjoint Health Research Ethics Board (REB15-312) [22].

### Participants

Participants with JIA were recruited by their clinician between July 2016 and November 2017 in collaboration with two pediatric rheumatology outpatient clinics [22]. Inclusion criteria were: 10–20 years old, a diagnosis of JIA, experiencing knee joint involvement (with or without other joint involvement other than the ankle), and active or inactive disease at time of testing [22]. Participants with JIA were excluded if systemic symptoms were present, if changes in medication occurred within the last three weeks, or if they had active ankle involvement [22]. We included knee involvement and excluded ankle involvement to assess knee joint biomechanics in previous studies [5, 7]. Participants with JIA were age and sex matched (within 18 months) with TD controls who were recruited via an online research portal by convenience [22]. Exclusion criteria for all participants included: pregnancy, diagnosis of other arthritides, lower extremity musculoskeletal injury within the past three months prior to testing that resulted in time loss from work, school, or sport, and contraindications as assessed through the Physical Activity Readiness Questionnaire for Everyone [22]. We conducted a sample size estimation using G*Power software [23] based on total body aBMD (g/cm2) by DXA for individuals with JIA and TD controls by Brabnikova Maresova et al. [JIA group mean (SD) 1.07 (0.19), TD group 1.21 (0.08)] [24]. Based on a paired t-test, due to the paired study design, and assuming a correlation between groups of 0.5, this equates to an effect size of 0.85 and a sample size of at least 17 pairs for a significance level of 0.05 and a power of 90%.

### Measurements

Data were collected in two sessions, one week apart [22]. Measurements included: anthropometrics (height, weight, and leg length), disease activity, and functional performance through right leg triple-single-leg-hop distance normalized to leg length (TSLH, three maximal consecutive hops forward with one leg– the distance measured being from the starting line to the point the heel lands on the third hop). Pain was assessed using the Child Health Assessment Questionnaire (CHAQ), which uses a visual analogue scale for disease-related pain and is converted into a continuous score of 0 to 3 [25]. Physical activity was measured using accelerometry (ActiGraph GT3X+, ActiGraph Inc., USA) with a 10-second epoch and worn for seven days including at least one weekend day [22]. Data were analyzed using ActiLife (v6.13.3, ActiGraph Inc.) and MVPA (minutes/day) was defined using the Evenson cut points as ≥ 2296 counts/minute [22]. Wear time was validated using the Choi algorithm [26] and data were included if participants wore the accelerometers for at least 10 waking hours per day on at least 5 days, including at least 1 weekend day [22]. Total body DXA (QDR 4500 A, Hologic Inc., USA) measured BMC, aBMD, and lean mass [22] with calibration procedures following the official recommendations of the International Society of Clinical Densitometry [27]. Height adjusted z-scores (HAZ) for BMC and aBMD were calculated as described by Zemel et al. [28]. In brief, sex-specific z-scores for BMC and aBMD were calculated relative to age from a reference dataset [28] and were then adjusted for height z-score using the Centre for Disease Control growth data [29]. Maturity offset (years from age at peak height velocity) was estimated using the approach described by Moore et al. [30]. To calculate height adjusted z-scores and maturity offset, exact chronological age was used.

### Data analysis

Participants with valid DXA and accelerometry data were included in analyses. R software was used to perform statistical analyses (2023.03.1 + 446, R Core Team, Austria). We summarized participant data by group and sex using median (min, max). We assessed group differences in participant characteristics and bone outcomes using multiple linear mixed effects models using the LMER package [31]. Base model covariates included group, sex, and maturity offset, except for the model with maturity offset as the dependent variable which was only adjusted for group and sex. Subsequent models evaluated the additional contributions of MVPA, TSLH max, and total body lean mass. We assessed model assumptions of normality of residuals using QQ plots and plots of residuals against fitted values. Significance was set at *p* < 0.05. We explored interactions between covariates, including effect modification by pain; however, none were significant; thus, we only retained models without interactions. Participant clusters based on sex and age matched pairs were considered as random effects.

## Results

### Participant characteristics

Of 32 initial participants with JIA, a subset of 21 (*n* = 7 males, *n* = 14 females) had valid DXA and accelerometry measures and were age and sex-matched with TD adolescents (Table 1). We excluded 11 of the 32 participants with JIA due to incomplete DXA data (*n* = 3), incomplete accelerometry data (*n* = 4), or no age and sex matched pair (*n* = 4). Adolescents with JIA were diagnosed between 0.0 and 3.3 years before assessment with a median of 1.2 years since diagnosis. Oligoarthritis was the most prevalent type of JIA in this sample (*n* = 12), followed by polyarticular arthritis (*n* = 7), and enthesis related arthritis (*n* = 2). 80% of participants with JIA for which medication data were collected (missing data for 1 participant) used at least two different classes of arthritis medications including: corticosteroids, biologics, disease-modifying antirheumatic drugs (DMARDs), and non-steroidal anti-inflammatory drugs (NSAIDS). Adolescents with JIA had a median of zero joints affected and range of motion impaired (range 0–3) and low physician global assessment of disease activity [female, *n* = 12, median 0.0 (0.0–1.0); male, *n* = 6, 0.6 (range 0.0-2.5) out of 10] and parent global assessment of disease activity [female, *n* = 10, 0.2 (0.0–2.0); male, *n* = 3, 1.7 (0.0–8.0) out of 10].Pain ranged from 0 to 2.3 [JIA, *n* = 21, median 0.15 (range 0.0-2.3); TD, *n* = 21, 0.0 (0.0 to 2.0) out of 3]. No differences between groups were observed for height [B (95% CI); 0.8 (-2.4 to 4.0) cm], body mass [0.3, (-5.5 to 6.2) kg), or maturity offset [-0.1, (-1.8 to 1.7) years].

### Bone mineral content and density

**Fig. 1:**
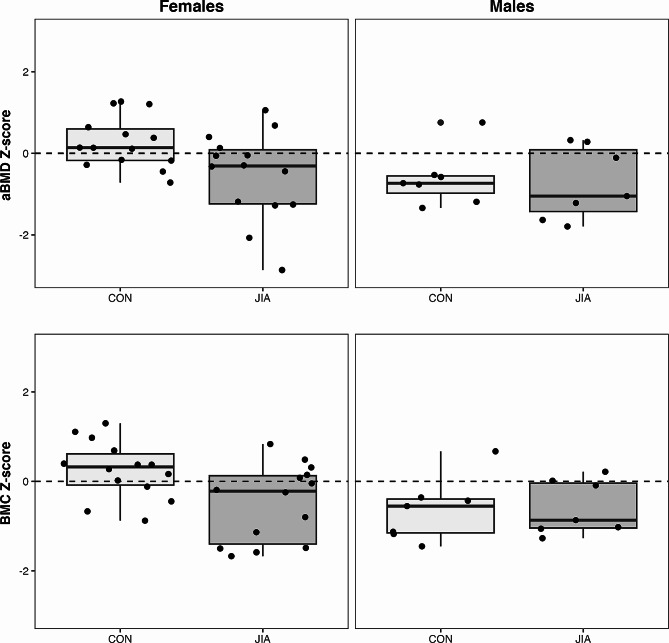
Bone outcomes by group and sex: aBMD (top), BMC (bottom), females (left), males (right). aBMD = areal bone mineral density, BMC = bone mineral content, CON = typically developing controls

Adolescents with JIA had lower unadjusted aBMD [β (95% CI); -0.04 (-0.08 to -0.002) g/cm^2^, *p* = 0.04] and HAZ aBMD compared with their TD peers [β (95% CI); -0.58 (-1.10 to -0.07), *p* = 0.03 (Fig. 1; Table 2). Adolescents with JIA also had lower HAZ BMC compared with their TD peers [-0.47 (-0.91 to -0.03), *p* = 0.04] (Fig. 1; Table 2), but not unadjusted for height BMC (-1 (-225 to 43) g, *p* = 0.18). Two adolescents with JIA had low HAZ aBMD (z-score < -2.0). All participants had HAZ BMC within the normal range compared with reference data (z-score > -2.0).

### MVPA, lean Mass, and TSLH

**Fig. 2:**
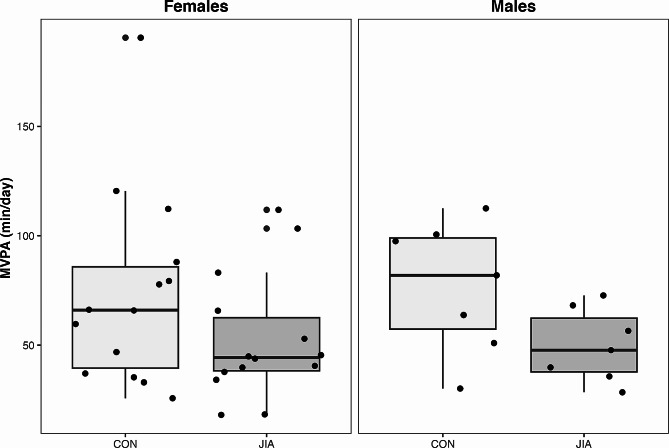
Moderate-to-vigorous physical activity (MVPA; min/day) by group for females (left) and males (right). CON = typically developing controls

Adolescents with JIA engaged in 22 min less MVPA day than their TD peers [-22.0 (-38.7 to -5.3) min, *p* = 0.01] (Fig. 2). However, MVPA was not associated with either HAZ aBMD [-0.01 (-0.01 to 0.01) g/cm^2^, *p* = 0.32] or HAZ BMC [0.00 (-0.01 to 0.00) g, *p* = 0.39]. No differences between groups were observed for lean mass [-0.4, (-3.2 to 2.5) kg or TSLH [-10 (-61 to 42) % leg length]. Lean mass was positively associated with both HAZ aBMD [0.05 (0.01 to 0.09) g/cm^2^, *p* = 0.03] and HAZ BMC [0.06 (0.03 to 0.10) g, *p* < 0.001] (Table 2). TSLH was positively associated with HAZ aBMD [0.00 (0.00 to 0.01) g/cm^2^, *p* = 0.04] (Table 2) and a similar trend was indicated in HAZ BMC [0.00 (-0.00 to 0.00) g, *p* = 0.09] (Table 2).

**Table 1:** Participant and bone characteristics by sex and group

	JIA Male (*n* = 7)			TD Male (*n* = 7)		JIA Female (*n* = 14)		TD Female (*n* = 14)
*Participant Characteristics*								
Age (years)	14.9 (13.3, 19.0)			14.6 (11.8, 18.1)		14.8 (10.7, 20.1)		15.5 (10.1, 19.9)
Maturity offset (years from age at peak height velocity)	1.7 (-0.2, 4.1)			0.9 (-1.8, 3.7)		2.4 (-1.9, 5.6)		2.3 (-1.7, 5.3)
Weight (kg)	61.0 (48.5, 101.5)			51.0 (34.0, 77.0)		50.3 (28.5, 66.0)		52.5 (34.00, 87.0)
Height (cm)	175.0 (160.3, 184.2)			170.2 (144.6, 182.5)		159.5 (133.5,175.0)		161.8 (143.8, 175.0)
*Total Body Bone Characteristics*								
aBMD (g/cm^2^)	1.0 (0.9, 1.2)			0.9 (0.8, 1.2)		1.0 (0.6, 1.1)		1.1 (0.8, 1.2)
BMC (g)	2070 (1381, 2963)			1649 (1160, 2652)		1752 (845, 2271)		1959 (1253, 2440)
aBMD height adjusted z-score	-1.1 (-1.8, 0.3)			-0.7 (-1.3, 0.8)		-0.3 (-2.9, 1.1)		0.1 (-0.7, 1.3)
BMC height adjusted z-score	-0.9 (-1.3, 0.2)			-0.6 (-1.5, 0.7)		-0.2 (-1.7, 0.8)		0.3 (-0.9, 1.3)
*Physical Activity and Muscle*								
MVPA (min/day)	47.7 (28.4, 72.7)			81.9 (30.1, 112.5)		44.3 (18.1, 111.9)		66.0 (25.7, 190.6)
TSLH **(**% of leg length)	499 (336, 649)			558 (461, 697)		500 (333, 643)		485 (380, 612)
Total body lean mass (kg)	53.5 (41.0, 61.5)			43.4 (28.4, 65.1)		36.6 (19.9, 47.7)		39.1 (26.1, 54.9)

*Data are presented as median (range). aBMD = areal bone mineral density, BMC = bone mineral content, MVPA = moderate-vigorous physical activity, TSLH = triple single leg hop.*

**Table 2:** Summary of linear mixed effect model analyses of bone outcomes

	β	95% CI		*p*-value	β	95% CI			*p*-value
*HAZ BMC*					*HAZ aBMD*				
Group	-0.47	(-0.91 to -0.03)		0.04*	-0.58	(-1.10 to -0.07)			0.03*
Sex	-0.51	(-0.98 to -0.04)		0.04*	-0.54	(-1.08 to 0.01)			0.06
MO (years)	0.04	(-0.15 to 0.07)		0.47	0.03	(-0.09 to 0.16)			0.59
*HAZ BMC*					*HAZ aBMD*				
Group	-0.55	(-1.02 to -0.08)		0.03*	-0.69	(-1.24 to -0.14)			0.02*
Sex	-0.52	(-0.99 to -0.05)		0.03*	-0.55	(-1.10 to -0.01)			0.047*
MO	-0.07	(-0.20 to 0.06)		0.28	-0.01	(-0.16 to 0.14)			0.93
MVPA (minutes/day)	-3.55*10^− 3^	(-0.01 to 0.00)		0.39	-0.01	(-0.01 to 0.01)			0.32
*HAZ BMC*					*HAZ aBMD*				
Group	-0.45	(-0.83 to -0.06)		0.02*	-0.57	(-1.05 to -0.08)			0.03*
Sex	-1.29	(-1.88 to -0.70)		< 0.001*	-1.13	(-1.88 to -0.39)			0.004*
MO	-0.26	(-0.41 to -0.11)		0.001*	-0.13	(-0.32 to 0.06)			0.18
Lean mass (kg)	0.06	(0.03 to 0.10)		< 0.001*	0.05	(0.01 to 0.09)			0.03*
*HAZ BMC*					*HAZ aBMD*				
Group	-0.45	(-0.88 to -0.02)		0.04*	-0.55	(-1.04 to -0.07)			0.03*
Sex	-0.61	(-1.07 to -0.14)		0.01*	-0.67	(-1.21 to -0.14)			0.02*
MO	-0.05	(-0.15 to 0.06)		0.38	0.02	(-0.10 to 0.15)			0.70
TSLH (% leg length)	2.24*10^− 3^	(-0.00 to 0.00)		0.09	0.00	(0.00 to 0.01)			0.04*

* p < 0.05; *Reference sex is girls and reference group is typically-developing controls. β = Beta coefficient, HAZ = Height adjusted z-scores, BMC = bone mineral content, aBMD = areal bone mineral density, MO = maturity offset.*

## Discussion

Participants with JIA in this study had lower HAZ aBMD and HAZ BMC compared to age and sex matched peers. Our findings are supported by several studies in adolescents with JIA [32–34]. Despite individuals with JIA having lower HAZ aBMD and HAZ BMC than their TD counterparts, most participants with JIA had HAZ aBMD and HAZ BMC values within a healthy range (95% and 100%, respectively). This concurs with findings from Galindo Zavala and colleagues who found that fewer than 5% of children and adolescents with JIA experience low BMC and aBMD [35]. In our study cohort, this may have been due to low joint involvement and range of motion impairment (94% of participants had zero or only one joint with impaired ROM), as the relatively good disease status of participants likely facilitated bone accrual.

### MVPA and bone outcomes

Participants with JIA performed substantially less MVPA per day than adolescents in the control group, which is consistent with other reports in youth with JIA [36]. Considering the relatively good disease status of the cohort, it was interesting that we observed such large group discrepancies in MVPA. Canadian physical activity guidelines recommend that youth attain 60 min per day of MVPA [37]. Only 29% of adolescents with JIA achieved this participation compared with 62% of TD adolescents in our cohort. The proportion of TD adolescents in our study who achieved the recommended daily physical activity is comparable to the 51% of Canadian youth achieving 60 min per day of MVPA pre-pandemic [38]. Individuals with JIA experience many barriers to movement that may influence lifetime bone accrual and other health benefits associated with physical activity, such as reduced pain and improved emotional well-being [16, 39]. It may be important to consider barriers that children and adolescents with JIA face that prevent physical activity participation, including joint pain and fear of being in pain, fatigue, embarrassment about not being able to participate in sport fully, and lack of accommodations to reduce anxiety around movement [3, 40]. Effective education strategies for children with chronic disease (and their caregivers) are needed to provide the tools to become more physically active.

It was surprising that despite group differences in MVPA and in contrast to our hypothesis, we did not observe a relationship between MVPA and HAZ aBMD or HAZ BMC. However, bone accrual is complex and influenced by several factors other than physical activity, including genetics, the endocrine environment, pharmacotherapy, and inflammation. For example, in patients with JIA, synovial macrophages produce inflammatory cytokines which increase production and activation of osteoclasts and leads to bone resorption [41]. It is possible that participants engaged in other forms of bone strengthening activities that may not have been captured by accelerometry. For example, a recent 3-month supervised lumbar spine and pelvic-core strengthening and stability program in conjunction with physical therapy (e.g., isometric strength, weight bearing, stretching, range of motion exercises) significantly improved BMC and aBMD of the femoral neck and lumbar spine compared with the control group which received only conventional physical therapy [4]. Likely neither these types of lumbar spine nor pelvic-core strengthening activities would be captured as MVPA via accelerometry as accelerometers are typically worn around the waist which is stationary and would not detect vertical acceleration during these types of exercises. It is also possible that participants with JIA decreased their MVPA after experiencing the onset of arthritis symptoms. As median years since diagnosis was short (1.2 years), a longer duration of disease may be needed to detect changes in bone health in relation to decreased MVPA. Longitudinal studies examining changes in MVPA would allow for a better understanding of the effect of physical activity on bone health in children and adolescents with JIA.

### Lean mass, functional performance, and bone outcomes

Muscle mass and strength are important determinants of bone accrual [19] and TSLH is a functional test that reflects muscular strength and power of the lower limbs [42]. Consistent with the functional muscle-bone unit theory [43], we found that both greater lean mass and TSLH were related to accruing greater HAZ aBMD and HAZ BMC. Our findings reflect current literature showing that lean mass is a correlate of greater absolute and z-score aBMD in adolescents with JIA [44]. The relationships we observed between lean mass and HAZ aBMD and HAZ BMC are weaker than previously reported in children and adolescents with JIA [4]. We suspect this is because our bone outcomes were adjusted for height (body size) in addition to lean body mass (another surrogate for body size). The taller an individual is, the more lean mass and bone mass they likely have [45, 46]. As most previous studies of bone health in adolescents with JIA did not adjust for height, lean mass would have been a stronger surrogate for body size than it was in our analyses.

### Strengths and limitations

Strengths of our study include assessment of device-based physical activity and adjusting DXA bone outcomes for age and height. We acknowledge several limitations of our study. Our study sample was small and total body DXA data were collected opposed to total body less head [22], which is recommended by the International Society for Clinical Densitometry [27] due to the change in contribution of the skull to aBMD and BMC during growth [28]. Given the age-matched nature of the study, we suspect differences in bone mass (with head vs. no head) did not bias findings. A primary limitation of DXA is that it is a two-dimensional assessment and cannot account for bone depth. Therefore, aBMD is systematically underestimated in smaller individuals [47]. Adjusting bone outcomes for height helps alleviate these limitations. Future work should consider a three-dimensional imaging modality, such as peripheral quantitative computed tomography (pQCT). Further, we did not collect data regarding dose or duration of medication or duration of active disease. Finally, due to the small sample size, we were unable to stratify the participants into specific subtypes of JIA or active vs. inactive disease status. While a strength of this study is considering both height adjusted z-scores and maturity offset to minimize the influence of body size and maturation on bone outcomes, a limitation is that race/ethnicity data were not collected so all participants were designated as white for height adjusted z-score calculations [22]. Z-scores may have differed slightly from those reported if participants were not white. Future studies should account for race/ethnicity and the lack of normative DXA data beyond white and black ethnicities must be addressed.

## Conclusion

Adolescents with JIA had significant deficits in bone outcomes compared with their TD peers. Despite substantial group differences in MVPA participation, we did not see a relationship between physical activity and bone outcomes. We found that adolescents who had more lean mass also had greater bone accrual. Physical activity promotes physical, emotional, and mental well-being and can prevent secondary consequences later in life; thus, physical activity should still be encouraged for children and adolescents with JIA as it is for their healthy peers.

## Data Availability

The data analysed during the current study are only available from the corresponding author on reasonable request.

## Abbreviations

JIA: juvenile idiopathic arthritis
aBMD: areal bone mineral density
BMD: bone mineral density
BMC: bone mineral content
TD: typically developing
TSLH: triple single leg hop
HAZ: height adjusted z-scores
DXA: dual X-ray absorptiometry
MVPA: moderate-vigorous physical activity
DMARDs: disease-modifying antirheumatic drugs
NSAIDs: non-steroidal anti-inflammatory drugs
